# Changes in Umami-Enhancing Nucleotides in White Mullet (*Ophiocephalus argus* var. Kimnra) Meat Stored at Ice Temperature

**DOI:** 10.3390/foods14173022

**Published:** 2025-08-28

**Authors:** Yin Zhang, Qing Li, Qing Zeng, Hongling Hu, Longyi Zhang, Li Dong, Jiao Zhou, Yuzhu Lin

**Affiliations:** 1 Meat Processing Key Laboratory of Sichuan Province, Chengdu University, Chengdu 610106, Chinahuhongling1221@163.com (H.H.); iidongli@163.com (L.D.); zzhoujiao2023@163.com (J.Z.); linyuzhu@stu.cdu.edu.cn (Y.L.); 2 College of Food and Biological Engineering, Chengdu University, Chengdu 610106, China

**Keywords:** ice-temperature storage, *Opniocepnalus argus* var. Kimnra, nucleotides, glycolysis

## Abstract

The aim of this study was to investigate the effect of ice-temperature (IT) storage on the umami-enhancing nucleotide content in white mullet (*Ophiocephalus argus* var. Kimnra) meat. White mullet dorsal muscle was used as the raw material, and 4 °C chilled storage was used as a reference. After determining the ice temperature (−0.6 °C) of the dorsal muscle, the effect of IT storage on its umami-enhancing nucleotides was investigated. The umami nucleotide levels, physicochemical properties (pH, muscle color, water-holding capacity, and cooking loss rate), glycolytic metabolites (lactic acid, pyruvic acid, and glycogen), and enzyme activities (lactate dehydrogenase, pyruvate kinase, and 5′-nucleotidase) in the dorsal muscle were examined. The results indicate that IT storage significantly (*p* < 0.05) lowered pH while improving the water-holding capacity compared to 4 °C chilled storage. Both storage conditions showed an initial increase followed by a decrease in the inosine 5′-monophosphate (IMP) content, while the content of guanosine 5′-monophosphate (GMP) progressively declined. IT storage maintained significantly (*p* < 0.05) higher IMP and GMP levels than chilled storage in the late storage stage. The accumulation of the bitter taste substances hypoxanthine (Hx), lactic acid, and pyruvic acid was reduced under IT storage. These findings demonstrate that IT storage effectively inhibits the degradation of umami-enhancing nucleotides and is beneficial for preserving the meaty taste of white mullet meat.

## 1. Introduction

Low-temperature storage is one of the most commonly used methods for preserving aquatic products, primarily including chilling refrigeration (0~10 °C); ice-temperature storage (0~−4 °C); and high- (−4~−6 °C), medium- (−6~−18 °C), low- (−18~−40 °C), and ultra-low-temperature freezing (<−40 °C) [1]. Among these, ice-temperature (IT) storage is a recently developed method, which involves storing food at a temperature near its freezing point, but the food is not frozen. Studies have found that IT storage helps inhibit the activity of endogenous enzymes, maintain cellular integrity, suppress the growth and reproduction of microorganisms, etc. [2]. Xu et al. [3] stored chicken breast meat at IT (−1 °C) and found that it could inhibit the total bacterial amount, delay its spoilage, and extend its shelf life to 13 days. IT (−1.5 °C) storage could also delay the decrease in the salt-soluble protein solubility, total sulfhydryl content, and sulfhydryl content in the chicken breast meat, and it inhibited the increase in surface hydrophobicity and the disulfide bond content [4]. Gu et al. [5] stored yak tenderloin at IT (−2 °C) and found that its color, water-holding capacity, and protein stability were superior to those of yak meat stored at chilling temperature (0 °C). Cao et al. [6] stored beef back meat at IT (−1.6 °C) and found that it effectively inhibited protein degradation and structural changes. Tian et al. [7] stored horse hind leg meat at IT (−1.51 °C) and found that it inhibited protein and lipid oxidation during the post-slaughter maturation process. Qin et al. [8] stored rainbow trout meat at IT (0 °C) and found that it effectively decreased the increase in pH, thereby inhibiting spoilage. Recently, Zhang et al. [9] stored pork bicipital muscle at IT (−1.4 °C) and found that it effectively inhibited the decrease in its umami-enhancing nucleotide content. Lee et al. [10] stored tuna filet in a supercooled condition (−3.5 °C) and found that the TVB-N value of the supercooled sample was lower than that of the chilled sample (4 °C). In addition, compared with the freeze–thaw sample, supercooling processing maintained the texture integrity of the product and reduced dripping. However, few studies have investigated the effect of IT storage on the umami-enhancing nucleotide content in fish muscle.

As the fifth basic taste, umami is a potential indicator of protein content in food [11]. The known nucleotides with umami-enhancing effects include inosine 5′-monophosphate (IMP), guanosine 5′-monophosphate (GMP), cytidine 5′-monophosphate (CMP), uridine 5′-monophosphate (UMP), xanthosine 5′-monophosphate (XMP), and adenosine 5′-monophosphate (AMP) [9,12]. Their metabolic pathways are as follows: adenosine triphosphate (ATP) in post-mortem muscle is broken down into adenosine diphosphate (ADP) and adenosine monophosphate (AMP) by endogenous enzymes, AMP loses its amino group to form inosine monophosphate (IMP), IMP loses its phosphate group to form hypoxanthine riboside (HxR) [13], and HxR loses its ribose to form hypoxanthine (Hx) [14]. Another pathway involves IMP connecting with a hydroxyl group to form xanthosine monophosphate (XMP), which is further decomposed into guanosine monophosphate (GMP). Among these nucleotides, IMP, GMP, CMP, UMP, and AMP have umami-enhancing effects [15], while HxR and Hx have a bitter taste. Studies have shown that 5′-nucleotidase (5′-NT) is the primary factor influencing the degradation of IMP in muscle [16]. Pacheco-Aguilar et al. [17] purified 5′-NT from the mantle of giant squid and found that it is also a key enzyme involved in AMP degradation during the ice storage of giant squid. Zhang et al. [16] prepared 5′-NT from white pork loin and evaluated its reaction conditions. These investigations provide a good basis for research on the effects of IT storage on the umami-enhancing nucleotide content in white mullet (*Ophiocephalus argus* var. Kimnra) meat.

White mullet became a nurtured freshwater fish species in China in 2022. It is characterized by a high potassium content and a low fat content [18]. Previous studies have investigated the types of heat-resistant bacteria in the mucus of white mullet [19], the antibacterial activity of bacterial strains in the mucus of white mullet [20], and the effects of carbon monoxide treatment on the quality of white mullet meat [21]. Additionally, some products have been developed, including instant soup powder [22], white mullet surimi cakes [23], fish flakes [24], and meat balls [25]. The white mullet is an endemic freshwater fish species in Sichuan Province, with its distribution and sales currently confined to the region. To enhance its market value and promote its commercial circulation, this study was conducted to investigate the preservation of the white mullet meat using ice-temperature storage. However, few studies have investigated the effect of IT storage on the umami-enhancing nucleotides in the muscle of white mullet, which are vital for the natural meaty taste of the meat. Therefore, the aim of this study was to investigate the effect of IT storage on the umami-enhancing nucleotide content in white mullet meat.

## 2. Materials and Methods

### 2.1. Materials

5′-IMP salt (HPLC ≥ 98%), 5′-GMP disodium salt hydrate (HPLC ≥ 98%), 5′-UMP disodium salt (HPLC ≥ 98%), 5′-CMP disodium salt (HPLC ≥ 98%), 5′-AMP disodium salt (HPLC ≥ 98%), 5′-IMP disodium salt (HPLC ≥ 98%), Hx (HPLC ≥ 98%), and HxR (HPLC ≥ 98%) were all purchased from Shanghai Yuanye Biotechnology Co., Ltd. (Shanghai, China) Ethanol was purchased from Fischer Chemical Company. Potassium dihydrogen phosphate, trichloroacetic acid (TCA), and other required chemical reagents were obtained from Tianjin Kemiou Chemical Reagent Co., Ltd. (Tianjin, China).

The white mullet used for sampling had an average weight of 500.0 ± 20.0 g and a body length of 25.0 ± 1.0 cm. Dorsal muscle meat was purchased from the Shiling Wholesale Market in Longquanyi District, Chengdu City; placed in a polyethylene foam box with ice cubes; and transported to our laboratory within 30 min for later use.

### 2.2. Methods

#### 2.2.1. Experimental Design

The fish back meat was cleaned and cut into 48 pieces with a size of 40 mm × 40 mm × 15 mm, of which 24 pieces were used for storage at IT, and the other 24 pieces were used for storage at 4 °C. Two DC−4015 low-temperature thermostatic baths (Shanghai Pingxuan Scientific Instrument Co., Ltd., Shanghai, China) were used for storage experiments. The storage temperatures were set at IT and 4 °C. The meat samples were sealed in polyethylene bags and placed in stainless boxes, and the boxes were stored in the DC-4015 low-temperature thermostatic bath. Samples were taken on the 0th, 1st, 2nd, 3rd, 4th, 5th, 6th, and 7th days of storage to measure the corresponding indicators.

#### 2.2.2. Measurement of IT

The IT of the white mullet dorsal muscle meat was determined according to the method of Zhang et al. [9]. The dorsal muscle was cleaned, cut into blocks of 20 mm × 20 mm × 15 mm, and wrapped in cling film. An AS887 four-channel temperature probe Shenzhen Huaqing Instrument Co., Ltd., Shenzhen, China) was inserted into the center of the white mullet meat and then placed in the DC-4015 low-temperature thermostatic bath to gradually cool the temperature down from 12.0 °C to −18.0 °C. Data was recorded once per min on a time–temperature curve, the freezing point is explicitly defined as the temperature value at which the cooling process exhibits a cessation of temperature drop and maintains a relative plateau, and the IT value was finally determined by recording the temperature changes in the white croaker meat and combining them with temperature curve data. Triplicate experiments were performed, and the average value was used for plotting.

#### 2.2.3. Determination of Nucleotide Levels

The levels of nucleotides (IMP, GMP, XMP, AMP, CMP, UMP, Hx, and HxR) in the fish meat were determined according to the method described by Zhang et al. [9]. K2025 high-performance liquid chromatography (HPLC, Wukong Instrument Co., Ltd., Shanghai, China) was used to determine the levels of nucleotides. Before determination, the muscle samples were frozen in liquid nitrogen for 10 min and then ground into powder using an A11 ultrafine grinder (IKA Company, Königsberg, Germany). A total of 10.0 g of the muscle powder was mixed with 40 mL of 5% (*w*/*v*) trichloroacetic acid solution and stood at 4 °C for 2 h. After centrifugation (5000× *g*, 10 min, 4 °C), the supernatant was transferred into a 100 mL glass beaker, and its pH was adjusted to 6.50 ± 0.05 with 3 mol/L potassium hydroxide solution. The solution was filtered with a medium-speed qualitative filter paper and made up to a volume of 50 mL in a volumetric flask. Finally, it was filtered through a 0.22 μm microporous membrane to prepare the chromatographic injection solution.

HPLC conditions: C18 reverse-phase chromatography column (4.6 mm × 250 mm, 5 μm); mobile phase: methanol, 0.05 mol/L potassium dihydrogen phosphate solution. The operating parameters of the chromatographic system include the following: a flow rate of 0.8 mL/min, a column temperature of 30 °C, and a UV detection wavelength of 250 nm. Triplicate determinations were performed, and the average values are reported.

#### 2.2.4. Determination of pH, Color, Water-Holding Capacity (WHC), and Cooking Loss Rate

##### PH

The pH value of each sample was measured using a Testo 205 handheld pH meter with temperature compensation (Testo Instrument International Trade (Shanghai) Co., Ltd., China) according to the method of Zhang [26]. A total of 5 g of minced meat was combined with 50 mL of deionized water, shaken, and then stood at room temperature (25.0 ± 2.0 °C) for 30 min. The pH of each sample was measured using a Testo 205 handheld pH meter with temperature compensation (Testo Instrument International Trade (Shanghai) Co., Ltd., Shanghai, China). The calibration of the pH probe was performed using buffer solutions with pH 4.00 and 6.86 at 25.0 ± 2.0 °C. The experiment was repeated three times in parallel.

##### Color

The color of the samples was measured according to the method of Cen [27]. The white mullet meat was cut into 30 mm × 20 mm × 15 mm cubes, and a 3nh color meter (Sanenchi Technology Co., Ltd., Shenzhen, China) was used for measurement. Prior to testing, the instrument was calibrated using a standard white plate. The color meter measured the color parameters of the samples, including the lightness value (L*), redness value (a*), and yellowness value (b*). For each sample, measurements were taken at five randomly selected locations. The measurement was repeated six times for each sample, and the mean value was reported.

##### Water-Holding Capacity (WHC)

The water-holding capacity of the white mullet meat was determined using the centrifugal method of Cai [28]. A total of 5 g of meat sample was weighed, wrapped in double-layer filter paper, placed in a TGL-1650 high-speed refrigerated centrifuge (Shuke Instrument Co., Ltd., Chengdu, China), and centrifuged at 3000 r/min at 4 °C for 15 min. The mass of the sample was measured to obtain the moisture content released after centrifugation. The experiment was repeated three times in parallel.

##### Cooking Loss Rate

The steam cooking loss rate of the white mullet meat was determined using the steam cooking method [29]. The white mullet meat was cut into 30 mm × 20 mm × 15 mm cubes, weighed, placed in a steaming bag, sealed, and then heated in an 85 °C water bath for 10 min. The samples were then cooled at room temperature (26 °C) and dried with filter paper to remove surface moisture. The cooking loss rate was calculated by measuring the reduced mass of the sample. The experiment was repeated three times in parallel.

#### 2.2.5. Determination of 5′-NT Activity

The 5′-NT activity in the fish meat was determined according to the method described by Zhang et al. [9]. An A041-2-1 5′-NT detection kit (Nanjing Jiancheng Bioengineering Research Institute, Nanjing, China) was used, and the activity units were expressed as U·g·protein^−1^. Triplicate determinations were performed, and the average value is reported.

#### 2.2.6. Determination of Glycogen, Pyruvate, and Lactic Acid (LD)

The levels of glycogen, pyruvate, and lactic acid (LD) in the white mullet muscle were determined according to the method of Zhang et al. [9], using A043-1-1 glycogen, A081-1-1 pyruvate, and A019-2-1 lactate detection kits (Nanjing Jiancheng Bioengineering Research Institute, China), respectively. Triplicate determinations were performed, and the mean values are reported.

#### 2.2.7. Determination of Pyruvate Kinase (PK) and Lactate Dehydrogenase (LDH) Levels

The levels of pyruvate kinase (PK) and total lactate dehydrogenase (LDH) in the white mullet meat were determined according to the method of Zhang et al. [9], using A076-1-1 pyruvate kinase and A020-2 lactate dehydrogenase detection kits (Nanjing Jiancheng Bioengineering Research Institute, China), respectively. Triplicate determinations were performed, and the average values are reported.

#### 2.2.8. Data Analysis

All experiments were conducted with three or more replicates, and the experimental data were analyzed using IBM SPSS 25.0 software (International Business Machines Corporation, New York, NY, USA). The data were analyzed by a one-way ANOVA test, and Fisher’s LSD test was used to evaluate the significant difference between the data, where significance was set as *p* < 0.05. The results are expressed as the mean ± SD. A Pearson correlation analysis was conducted, with “*”indicating correlation at the *p* < 0.05 level and “**”indicating correlation at the *p* < 0.01 level. Graphs were created using GraphPad Prism 8 software (GraphPad Software, LLC, San Diego, CA, USA).

## 3. Results and Analysis

### 3.1. Measurement Results of Ice Temperature

**Figure 1:**
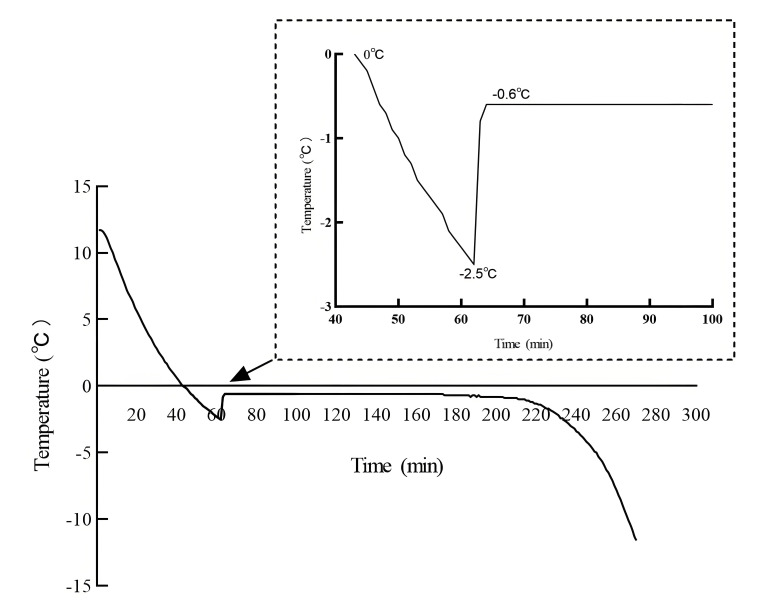
Freezing curve of white mullet meat.

A freezing curve of the white mullet meat was determined to accurately measure its IT (Figure 1). The data in Figure 1 show a temperature inflection at 63 min, where the center temperature of the fish meat reaches −2.5 °C, then increases to −0.6 °C, and remains at −0.6 °C for an extended period. Therefore, the IT of the dorsal muscle is −0.6 °C, which is higher than that of pork ham bicipital muscle (−1.4 °C) [9] but similar to that of Atlantic cod back muscle (−0.9 °C) [30].

IT is influenced by factors such as muscle type, location, tissue structure, and moisture content, all of which affect muscle thermal conductivity [31]. As fish meat contains a high moisture content of 70–80%, with most of it being free water, the freezing point is relatively high at approximately −0.5 °C to −2.5 °C [32]. This might be the reason why the IT of the dorsal muscle is higher than that of ham bicipital muscle and similar to that of Atlantic cod back muscle.

### 3.2. Nucleotides

#### 3.2.1. Effect of Storage Temperature on Nucleotide Content

The changes in the nucleotide content in the white mullet meat stored at IT (−0.6 °C) and 4 °C are shown in Table 1. The data in Table 1 show that the AMP content in the IT and 4 °C groups decreased significantly (*p* < 0.05) from day zero to the second day and then increased, with no significant (*p* > 0.05) changes after the second day. The AMP content in the 4 °C group was higher than that in the IT group on the first day but lower than that in the IT group from the fourth to seventh days. The IMP content in the IT and 4 °C groups increased significantly (*p* < 0.05) from day zero to the first day; however, that in the IT group kept increasing until the third day, whereas that in the 4 °C group showed fluctuating changes. The IMP content in the IT and 4 °C groups decreased significantly (*p* < 0.05) after the third day, although it remained higher in the IT group than in the 4 °C group after the first day. The HxR and Hx contents in the IT and 4 °C groups increased significantly (*p* < 0.05) after the first day, although the Hx content in the IT group remained lower than that in the 4 °C group after the first day. Regarding the HxR content, it was lower in the IT group than in the 4 °C group from the first to the fourth day, similar between the two groups on the fifth and sixth days, and higher in the IT group than in the 4 °C group on the seventh day. The XMP content in the IT and 4 °C groups decreased on the second day; however, it remained stable in the IT group from the first to the third day, whereas it kept decreasing in the 4 °C group from the first to the second day and then increased significantly (*p* < 0.05) from the second to the seventh day. Furthermore, it increased significantly (*p* < 0.05) in the IT group on the fourth day and then showed no significant (*p* > 0.05) changes, and it was lower in the IT group than in the 4 °C group on the sixth and seventh days. The GMP content in the IT and 4 °C groups decreased significantly (*p* < 0.05) from day zero to the second day and then remained stable from the third to the seventh day; it was significantly (*p* < 0.05) lower in the IT group than in the 4 °C group on the first and second days, but it was significantly (*p* < 0.05) higher in the IT group than in the 4 °C group on the seventh day. The UMP content in the IT group increased significantly (*p* < 0.05) from the first to the third day and then fluctuated up and down. The UMP content in the 4 °C group increased significantly (*p* < 0.05) from day zero to the second day and then decreased continuously.

**Figure 2:**
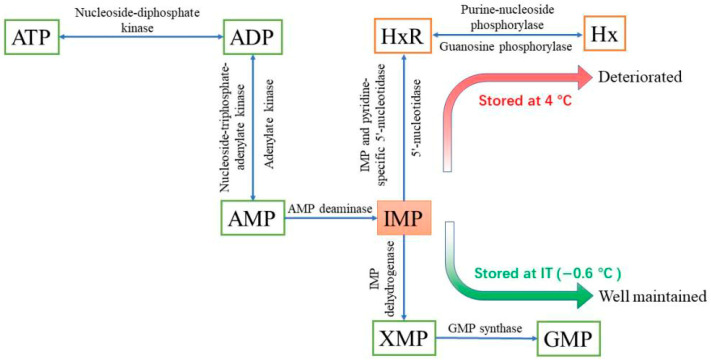
Conceptual diagram of nucleotide metabolic changes.

The metabolic pathways of the nucleotides and their effects on the white mullet meat are shown in Figure 2. AMP is generated from ATP in post-mortem muscle by endogenous enzymes. Low-temperature storage can slow down the metabolic rate of ATP to AMP [33], IT is lower than 4 °C, and a lower storage temperature extends the metabolic time of ATP to AMP. Thus, these factors might be the reason why the AMP content in the IT and 4 °C groups decreased significantly (*p* < 0.05) from day zero to the second day and then increased, with no significant (*p* > 0.05) changes after the second day. The AMP content in the 4 °C group was higher than that in the IT group on the first day but lower than that in the IT group from the fourth to the seventh day. AMP loses its amino group to form IMP, and IMP loses its phosphate group to form HxR or connects with a hydroxyl group to form XMP. A higher storage temperature accelerates the metabolism of IMP into HxR and Hx [34]. This might be the reason why the IMP content in the 4 °C group was lower than that in the IT group from the first to the seventh day and why the HxR and Hx contents in the 4 °C group were higher than those in the IT group from the first to the fifth and seventh days, respectively. More HxR was decomposed into Hx on the fifth, sixth, and seventh days in the 4 °C group, and this might be the reason why the HxR content in the 4 °C group was lower than that in the IT group on the same days. Hx has a bitter taste [35]. The result of the Hx content in the IT group being lower than that in the 4 °C group suggests that IT storage could inhibit the accumulation of the bitter taste substance Hx. GMP can be produced by XMP or decomposed from guanosine-5′-triphosphate (GTP) [36]. The trend in the GMP content is similar to that in the AMP content, thus suggesting that GTP contributed more to the production of GMP. In this case, IT being lower than 4 °C might be the reason why the GMP content in the IT group was lower than that in the 4 °C group on the first and second days and then changed similarly from the third day. UMP and CMP are generated by the release of energy from uridine-5′-triphosphate (UTP) and cytidine-5′-triphosphate (CTP), respectively. Similarly, IT being lower than 4 °C might be the reason why the UMP and CMP contents in the IT group were higher than those in the 4 °C group from the first to the seventh day.

#### 3.2.2. Correlation of Nucleotides

The Pearson correlation coefficients for the nucleotides are shown in Table 2. The results show that AMP was significantly (*p* < 0.05) correlated with IMP in the IT group but not significantly (*p* > 0.05) correlated with it in the 4 °C group. IMP was significantly (*p* < 0.05) correlated with XMP in the 4 °C group but not significantly (*p* > 0.05) correlated with it in the IT group. XMP was significantly (*p* < 0.05) correlated with GMP in the 4 °C group but not significantly (*p* > 0.05) correlated with it in the IT group. These correlations are inconsistent with nucleotide metabolic theory.

According to nucleotide metabolic theory, IMP is produced by AMP losing its amino group, IMP degrades into HxR and XMP, XMP further decomposes into GMP, and CMP is derived from UMP [13]. Therefore, there should have been high correlations between AMP and IMP, IMP and XMP, XMP and GMP, and UMP and CMP. However, AMP was significantly (*p* < 0.05) correlated with IMP in the IT group but not significantly (*p* > 0.05) correlated with it in the 4 °C group. IMP was significantly (*p* < 0.05) correlated with XMP in the 4 °C group but not significantly (*p* > 0.05) correlated with it in the IT group. XMP was significantly (*p* < 0.05) correlated with GMP in the 4 °C group but not significantly (*p* > 0.05) correlated with it in the IT group. These results suggest that the nucleotide metabolic process differs between the IT and 4 °C groups. To further confirm the differences, the physicochemical indices, glycolytic metabolites, and endogenous enzyme activities of the dorsal muscle were examined.

### 3.3. Effect of IT Storage on pH, WHC, Cooking Loss, and Color of White Mullet Meat

#### 3.3.1. PH, WHC, and Cooking Loss

**Figure 3:**
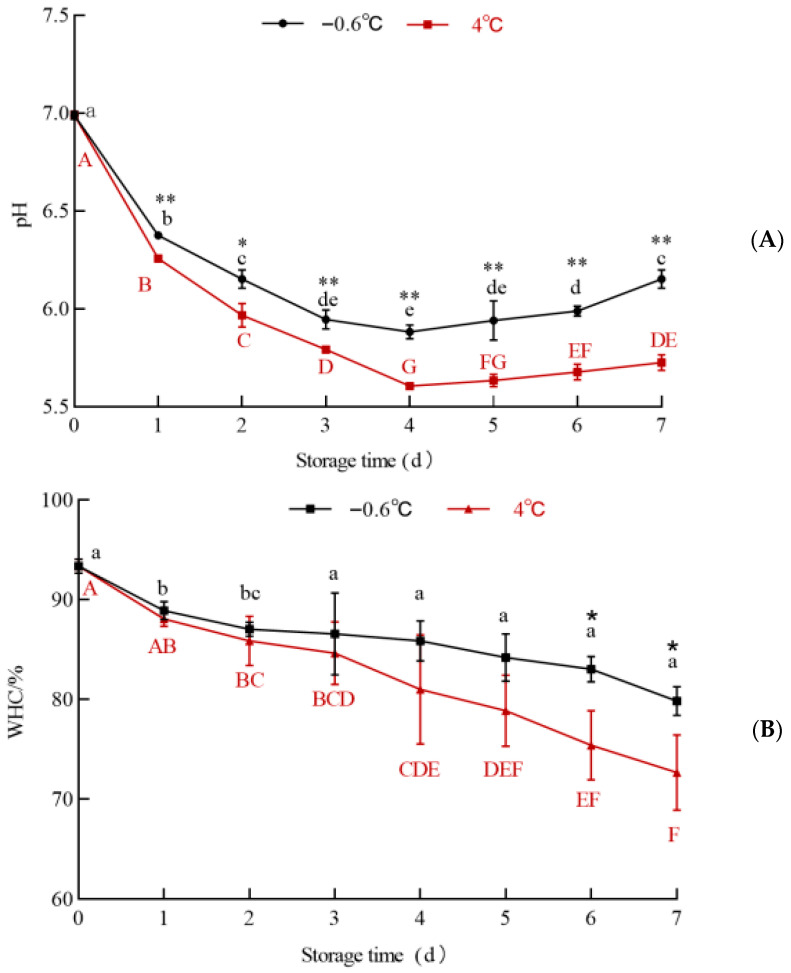

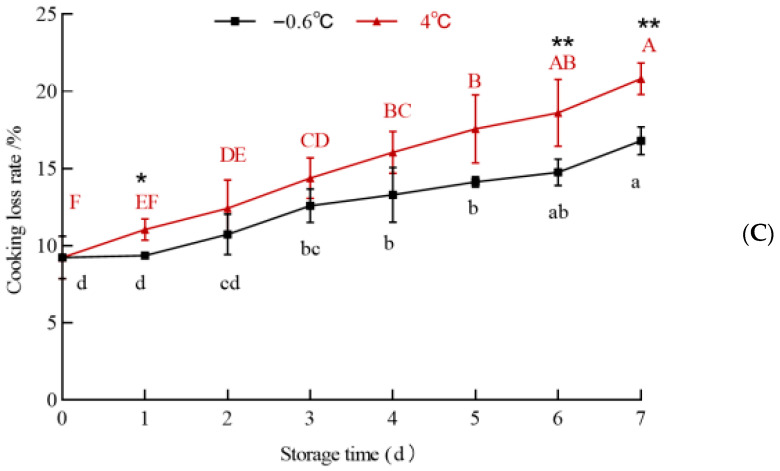
The effect of IT and chilled storage on the pH (**A**), WHC (**B**), and cooking loss (**C**) of the white mullet meat. The data are reported as the mean ± SD. The uppercase and lowercase letters above the points represent significant (*p* < 0.05) differences within the group. “*” indicating significant differences at the *p* < 0.05 level and “**” indicating significant differences at the *p* < 0.01 level.

The effect of IT and 4 °C storage on the pH of the white mullet meat is shown in Figure 3A. The data in Figure 3A show that the pH in the IT and 4 °C groups decreased significantly (*p* < 0.05) from the first day and then increased after reaching the lowest levels on the fourth day. The pH in the IT group was significantly (*p* < 0.05) higher than that in the 4 °C group from the first to the seventh day. A similar significant (*p* < 0.05) difference was shown in the WHC in the IT and 4 °C groups (Figure 3B), which is frequently used to indicate the damage extent of the muscle internal structure [37]. The cooking loss in the IT and 4 °C groups (Figure 3C) showed the opposite trend to the WHC (Figure 3B).

Generally, the lactic acid produced by glycogen aerobic oxidation after slaughter is the main factor that causes a decrease in muscle pH [38]. This suggests that lactic acid and glycogen content are the main factors that influence the pH in the IT and 4 °C groups. To confirm this hypothesis, the glycogen and lactic acid content in the IT and 4 °C groups was determined. A lower pH causes more damage to the muscle structure [39], thus resulting in a decrease in the WHC and an increase in cooking loss. This might be the reason why the WHC in the IT group showed a similar significant (*p <* 0.05) difference to pH and why the cooking loss in the IT and 4 °C groups showed the opposite trend to the WHC.

#### 3.3.2. Muscle Color

The effect of storage temperature on the color of the white mullet meat is shown in Table 3. The data show that the L* and b* values in the IT and 4 °C groups changed similarly, but the a* value changed differently: the a* value in the IT group on the sixth, seventh, and eighth days was significantly (*p* < 0.05) higher than that in the 4 °C group.

The hemoglobin content is a key factor of the muscle a* value [40]. Therefore, more hemoglobin might have been retained in the IT group on the sixth, seventh, and eighth days, leading to the a* value in the IT group on the same days being significantly (*p* < 0.05) higher than that in the 4 °C group.

### 3.4. 5′-NT Activity

**Figure 4:**
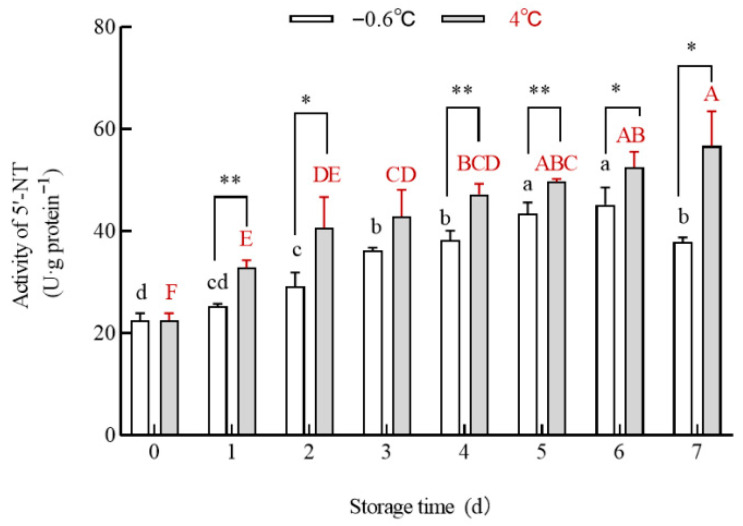
The effect of IT and chilled storage on the 5′-NT activity of the white mullet meat. The data are reported as the mean ± SD. The uppercase and lowercase letters above the bars represent significant (*p* < 0.05) differences within the group. “*” indicating significant differences at the *p* < 0.05 level and “**” indicating significant differences at the *p* < 0.01 level.

To confirm the contribution of 5′-NT to the IMP and HxR contents, the 5′-NT content in the IT and 4 °C groups was determined (Figure 4). The data in Figure 4 show that the 5′-NT activity in the white mullet meat in the 4 °C group was higher than that in the IT group from the first to the seventh day. The 5′-NT activity in the white mullet meat in the 4 °C group significantly (*p <* 0.05) increased with the storage time. Meanwhile, the 5′-NT activity in the white mullet meat in the IT group significantly (*p* < 0.05) increased from day zero to the sixth day and decreased significantly (*p* < 0.05) on the seventh day. The correlation coefficient between the 5′-NT activity and the IMP content was R^2^_4 °C_ = 0.886 and R^2^_−0.6 °C_ = 0.2771, respectively. The correlation coefficient between the 5′-NT activity and the HxR content was R^2^_4 °C_ = 0.9748 and R^2^_−0.6 °C_ = 0.8908, respectively.

**Figure 5:**
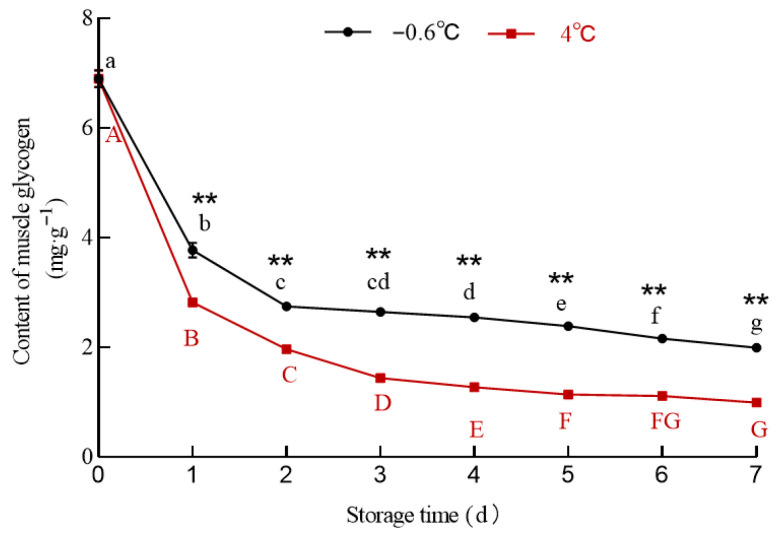
The effect of IT and chilled storage on the glycogen content of the white mullet meat. The data are reported as the mean ± SD. The uppercase and lowercase letters above the points represent significant (*p* < 0.05) differences within the group. “**” indicating significant differences at the *p* < 0.01 level.

**Figure 6:**
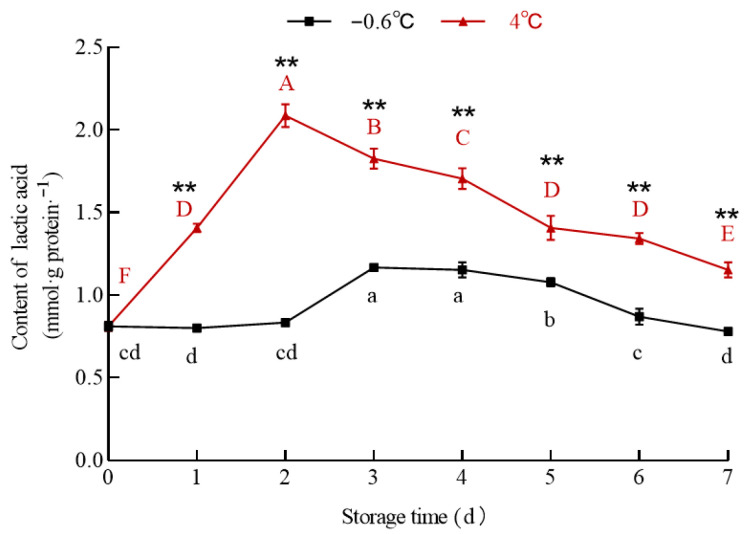
Effect of IT and chilled storage on lactic acid content of the white mullet meat. The data are reported as mean ± SD. The uppercase and lowercase letters above the points represent significant (*p* < 0.05) differences within the group. “*” indicating significant differences at the *p* < 0.01 level.

**Figure 7:**
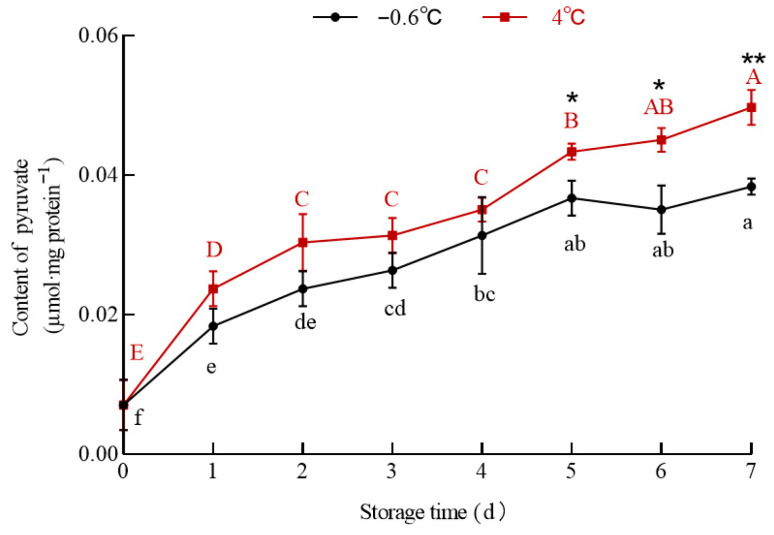
The effect of IT and chilled storage on the pyruvate content of the white mullet meat. The data are reported as the mean ± SD. The uppercase and lowercase letters above the points represent significant (*p* < 0.05) differences within the group. “*” indicating significant differences at the *p* < 0.05 level and “**” indicating significant differences at the *p* < 0.01 level.

**Figure 8:**
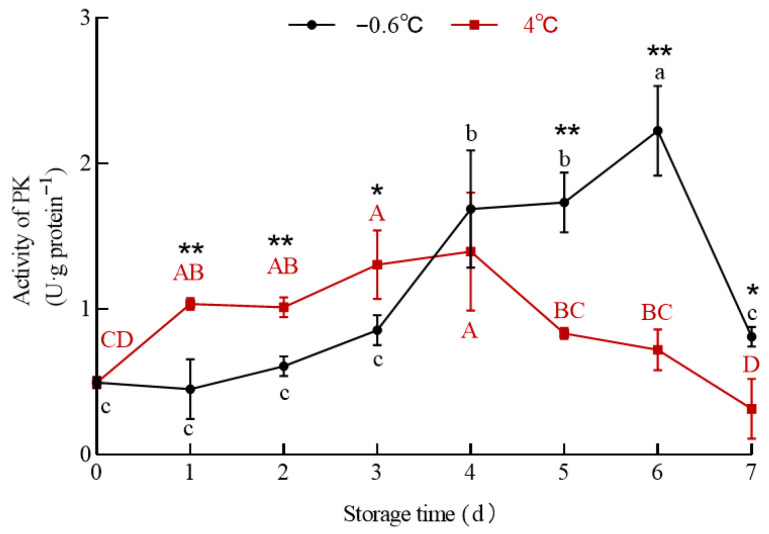
The effect of IT and chilled storage on the PK activity of the white mullet meat. The data are reported as the mean ± SD. The uppercase and lowercase letters above the points represent significant (*p* < 0.05) differences within the group. “*” indicating significant differences at the *p* < 0.05 level and “**” indicating significant differences at the *p* < 0.01 level.

**Figure 9:**
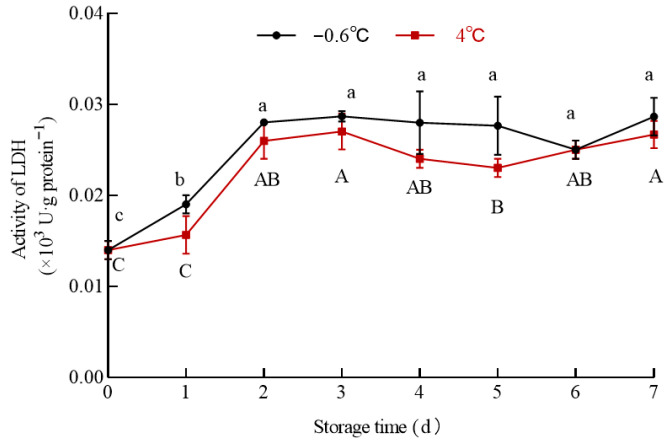
The effect of IT and chilled storage on the LDH activity of the white mullet meat. The data are reported as the mean ± SD. The uppercase and lowercase letters above the points represent significant (*p* < 0.05) differences within the group.

Endogenous 5′-NT is a key enzyme catalyzing the conversion of IMP to HxR [16]. The higher activity of 5′-NT catalyzes more IMP decomposed into HxR. As the 5′-NT activity in the IT group was lower than that in the 4 °C group, this might be the reason why the IMP content in the IT group was higher than that in the 4 °C group and why the HxR content in the IT group was lower than that in the 4 °C group from the first to the seventh day. The correlation coefficient between the 5′-NT activity and the IMP (R^2^_4 °C_ = 0.886 > R^2^_−0.6 °C_ = 0.2771) or HxR content (R^2^_4 °C_ = 0.9748 > R^2^_−0.6 °C_ = 0.8908) indicates that there is a higher correlation in the 4 °C group than in the IT group. This suggests that other factors might influence the conversion of IMP to HxR. To verify this speculation, the contents of glycogen (Figure 5), lactic acid (Figure 6), and pyruvate (Figure 7) and the activities of pyruvate kinase (Figure 8) and lactate dehydrogenase (Figure 9) were compared.

### 3.5. Glycogen, Lactic Acid (LD), and Pyruvate

#### 3.5.1. Glycogen

The changes in the glycogen content in the white mullet meat at IT and 4 °C storage are shown in Figure 5. The glycogen content in the white mullet meat was the highest (6.897 mg/g) at 0 d. With the increase in storage time, the glycogen content in both the IT and 4 °C groups decreased significantly (*p* < 0.05), although it was significantly (*p* < 0.05) higher in the IT group than in the 4 °C group from the first to the seventh day. This result is consistent with that of the pH being higher in the IT group than in the 4 °C group.

After fish slaughter, blood circulation stops, and oxygen supply is interrupted. At this time, muscle glycogen is broken down into lactic acid through anaerobic metabolism [41]. A higher storage temperature accelerates the anaerobic metabolism process [42]. This might be the main reason why the glycogen content decreased significantly (*p* < 0.05) with the increase in storage time and why the glycogen content in the IT group was higher than that in the IT group. To verify the contribution of lactic acid to the pH in the IT and 4 °C groups, the lactic acid content in the muscle was determined (Figure 6).

#### 3.5.2. Lactic Acid

The data in Figure 6 show that the lactic acid content in the white mullet meat in the IT and 4 °C groups changed differently. The lactic acid content in the 4 °C group increased significantly (*p* < 0.05) from day zero to the second day and then decreased significantly (*p* < 0.05) from the second to the seventh day. The lactic acid content in the IT group showed no significant (*p* > 0.05) change from day zero to the second day, increased significantly (*p* < 0.05) from the second to the third day, and then decreased significantly (*p* < 0.05) from the fourth to the seventh day. The correlation coefficients between the lactic acid content and pH in the 4 °C and IT groups were R^2^_4 °C_ = 0.7272 and R^2^_−0.6 °C_ = 0.4, respectively.

Lactic acid is a product of glycolysis. Under anaerobic conditions, organisms undergo lactic acid fermentation, in which glucose is converted into lactic acid via pyruvate under the catalysis of lactate dehydrogenase [43]. According to this theory, lactic acid should be the main factor influencing muscle pH, and there should be a high correlation between the lactic acid content and pH. However, the results show that the correlation between the lactic acid content and pH was high in the 4 °C group and weak in the IT group. This result suggests that besides lactic acid, there are other acidic substances that might affect the pH of the white mullet meat. To further identify other acidic substances influencing the pH in the IT group, the pyruvate content was determined (Figure 7).

#### 3.5.3. Pyruvate

The data in Figure 7 show that the pyruvate content in the white mullet meat in the IT and 4 °C groups increased significantly (*p* < 0.05) with the increase in storage time, but it was lower in the IT group than in the 4 °C group from the first to the seventh day. The correlation coefficients between the pyruvate content and pH in the 4 °C and IT groups were R^2^_4 °C_ = 0.9578 and R^2^_−0.6 °C_ = 0.9515, respectively. Comparing the correlation coefficients between the pH and lactic acid and the pH and pyruvate suggests that the pyruvate content was the main factor causing the change in muscle pH and not lactic acid.

Pyruvate is more acidic than lactic acid. The pyruvate content in the 4 °C group was higher than that in the IT group from the first to the seventh day, and there was a high correlation between the pyruvate content and pH. Thus, it can be speculated that lactic acid and pyruvate are the main factors causing the pH in the 4 °C group to be lower than that in the IT group from the first to the seventh day, with pyruvate being the main factor influencing the pH in the IT group. This speculation is supported by the fact that glycogen is broken down into pyruvate and then converted to lactic acid with the catalyzation of lactate dehydrogenase (LDH) under anaerobic conditions [44]. Under aerobic or specific metabolic demand conditions, lactic acid can generate pyruvate unidirectionally under the action of LDH [45]. To further identify the relationship between lactic acid, pyruvate, and endogenous metabolic enzymes, the activities of pyruvate kinase (PK) and LDH were determined.

### 3.6. PK and LDH Activities

#### 3.6.1. PK

The PK activity in the white mullet meat stored at IT and 4 °C is shown in Figure 8. The data in Figure 8 show that the PK activity in the IT and 4 °C groups changed differently. The PK activity in the IT group showed no significant (*p* > 0.05) increase from day zero to the third day, increased significantly (*p* < 0.05) from the third to the sixth day, and then decreased significantly (*p* < 0.05) from the sixth to the seventh day. The PK activity in the 4 °C group increased significantly (*p* < 0.05) from day zero to the fourth day and then decreased significantly (*p* < 0.05) from the fourth to the seventh day. This result is consistent with that of the pyruvate content in both the IT and the 4 °C groups increasing with the storage time and that of the pyruvate content being lower in the IT group than in the 4 °C group.

PK is one of the rate-limiting enzymes in the glycolytic pathway, and it catalyzes the irreversible conversion of phosphoenolpyruvate into pyruvate [46]. As a fish is slaughtered, the muscle glycogen content has a certain value. According to the kinetics of enzyme-catalyzed reactions, when the substrate content has a certain value, the reaction rate is the highest in the initial stage, and the product is rapidly generated; with the consumption of the substrate, the reaction rate gradually decreases, and the product concentration stabilizes. This is consistent with the decreasing trend in the glycogen content (Figure 5) and the increasing trend in the pyruvate content (Figure 7). Therefore, the limited glycogen content in the muscle might be the main factor influencing the PK activity in the IT and 4 °C groups.

#### 3.6.2. LDH

The LDH activity in the white mullet meat stored at IT and 4 °C is shown in Figure 9. The data in Figure 9 show that the LDH activity in both the IT and 4 °C groups exhibited a trend of initially increasing and then remaining stable. The LDH activity in the IT group was higher than that in the 4 °C group from the first to the seventh day. The correlation coefficients between the LDH and lactic acid content in the 4 °C and IT groups were R^2^_4 °C_ = 0.467 and R^2^_−0.6 °C_ = 0.2736, respectively. The correlation coefficient values suggest that there was a weak correlation between LDH and the lactic acid content.

According to the function of LDH, it catalyzes the conversion of pyruvate to lactic acid under anaerobic conditions [47]. Therefore, there should be a high correlation between LDH and the lactic acid content. However, the correlation between the LDH activity and the lactic acid content was weak. A possible reason for this unusual result might be that some lactic acid was converted into pyruvate during the storage process because, under aerobic or specific metabolic demand conditions, lactic acid can generate pyruvate unidirectionally under the action of LDH [48]. This conjecture is supported by the high correlation between the pyruvate content and pH in the 4 °C and IT groups.

## 4. Conclusions

The IT of white mullet dorsal muscle is −0.6 °C. IT storage significantly (*p* < 0.05) lowered the pH while improving the WHC of the dorsal muscle compared to 4 °C chilled storage. IT storage maintained significantly (*p* < 0.05) higher IMP and GMP levels than 4 °C storage in the late storage stage. The accumulation of the bitter taste substances Hx, lactic acid, and pyruvic acid was reduced under IT storage. These findings demonstrate that IT storage effectively inhibits the degradation of umami-enhancing nucleotides and is beneficial for preserving the meaty taste of white mullet meat. This provides technical support for maintaining the quality of white mullet meat during storage, transportation, and market circulation.

## Figures and Tables

**Table 1:** Changes in nucleoside content in the white mullet meat under different storage conditions.

Temperature (°C)	Time (d)	AMP	IMP	HxR	Hx	XMP	GMP	UMP	CMP
4	0	42.93 ± 8.36 ^a^	22.73 ± 0.01 ^e^	3.44 ± 0.03 ^h^	0.34 ± 0.05 ^h^	0.25 ± 0.01 ^d^	22.33 ± 0.01 ^a^	0.01 ± 0.00 ^e^	2.15 ± 0.73 ^a,b^
	1	35.64 ± 0.19 ^b,^*	45.54 ± 0.03 ^a^	25.69 ± 0.02 ^g,^*	3.33 ± 0.02 ^g,^*	0.19 ± 0.00 ^f^	20.28 ± 0.18 ^b,^*	0.16 ± 0.08 ^d^	2.61 ± 0.01 ^a^
	2	19.99 ± 0.15 ^c^	35.09 ± 0.06 ^c^	42.28 ± 0.01 ^f,^*	6.13 ± 0.02 ^f,^*	0.12 ± 0.01 ^g^	12.43 ± 0.30 ^c,^*	0.54 ± 0.00 ^a^	2.06 ± 0.05 ^a,b^
	3	21.86 ± 0.17 ^c^	45.70 ± 0.16 ^a^	44.40 ± 0.05 ^e,^*	8.59 ± 0.01 ^e,^*	0.22 ± 0.00 ^e^	10.28 ± 0.19 ^d,e^	0.34 ± 0.00 ^b^	2.05 ± 0.02 ^a,b^
	4	21.18 ± 0.32 ^c^	36.93 ± 0.17 ^b^	54.84 ± 0.24 ^b,^*	10.04 ± 0.02 ^d,^*	0.26 ± 0.00 ^d^	10.61 ± 0.16 ^d^	0.24 ± 0.00 ^c^	2.10 ± 0.02 ^a,b^
	5	21.33 ± 0.38 ^c^	29.10 ± 0.20 ^d^	53.10 ± 0.06 ^c^	11.23 ± 0.02 ^c,^*	0.28 ± 0.00 ^c^	9.99 ± 0.28 ^e^	0.19 ± 0.00 ^c,d^	2.03 ± 0.02 ^b^
	6	23.02 ± 0.43 ^c^	10.02 ± 0.04 ^f^	56.29 ± 0.08 ^a^	15.11 ± 0.02 ^b,^*	0.37 ± 0.00 ^b,^*	8.37 ± 0.38 ^g^	0.04 ± 0.00 ^e^	1.92 ± 0.02 ^b^
	7	21.89 ± 0.53 ^c^	4.98 ± 0.01 ^g^	50.23 ± 0.07 ^d^	18.26 ± 0.03 ^a,^*	0.499 ± 0.00 ^a,^*	8.98 ± 0.42 ^f^	0.00 ± 0.00 ^e^	1.96 ± 0.02 ^b^
−0.6	0	42.93 ± 8.36 ^a^	22.74 ± 0.01 ^g^	3.44 ± 0.00 ^h^	0.34 ± 0.05 ^h^	0.25 ± 0.01 ^a,b,c^	22.33 ± 0.01 ^a^	0.01 ± 0.00 ^b^	2.15 ± 0.73 ^a^
	1	24.62 ± 0.26 ^b^	61.48 ± 3.03 ^e,^*	16.08 ± 0.13 ^f^	1.46 ± 0.01 ^f^	0.17 ± 0.04 ^c^	15.27 ± 0.36 ^b^	0.09 ± 0.01 ^b^	2.53 ± 0.11 ^a^
	2	17.94 ± 4.78 ^b^	74.07 ± 2.35 ^c,^*	14.35 ± 0.12 ^g^	1.19 ± 0.01 ^g^	0.18 ± 0.012 ^b,c,^*	7.27 ± 0.34 ^e^	0.98 ± 0.85 ^a,^*	2.41 ± 0.02 ^a^
	3	22.18 ± 2.08 ^b^	115.93 ± 2.64 ^a,^*	32.62 ± 0.46 ^e^	2.81 ± 0.04 ^e^	0.20 ± 0.06 ^b,c^	10.77 ± 0.24 ^c^	1.32 ± 0.11 ^a,^*	2.31 ± 1.13 ^a^
	4	24.81 ± 4.39 ^b^	80.363 ± 1.372 ^b,^*	48.95 ± 0.52 ^d^	5.11 ± 0.04 ^d^	0.31 ± 0.02 ^a^	10.97 ± 0.35 ^c^	0.823 ± 0.11 ^a,^*	2.19 ± 0.08 ^a^
	5	23.73 ± 3.84 ^b^	69.20 ± 1.32 ^d,^*	57.90 ± 0.71 ^b,^*	6.84 ± 0.09 ^c^	0.27 ± 0.05 ^a,b^	10.84 ± 0.26 ^c,^*	0.96 ± 0.24 ^a,^*	2.24 ± 0.24 ^a^
	6	27.71 ± 9.68 ^b^	66.53 ± 0.73 ^d,^*	56.90 ± 0.42 ^c,^*	7.34 ± 0.32 ^b^	0.29 ± 0.01 ^a^	9.86 ± 0.78 ^d,^*	1.18 ± 0.05 ^a,^*	2.13 ± 0.01 ^a^
	7	23.28 ± 5.09 ^b^	41.15 ± 0.47 ^f,^*	61.89 ± 0.39 ^a,^*	10.07 ± 0.09 ^a^	0.26 ± 0.08 ^a,b,c^	10.77 ± 0.34 ^c,^*	1.01 ± 0.47 ^a,^*	2.06 ± 0.30 ^a^

Values expressed in mg/100 g. The data are reported as the mean ± SD. The lowercase letters represent significant (*p* < 0.05) differences within the group. “*” indicates the significance of intergroup differences at the *p* < 0.05 level.

**Table 2:** Pearson correlation analysis of nucleotides.

Temperature (°C)	Factors	AMP	IMP	HxR	Hx	XMP	GMP	UMP	CMP
4	AMP	1	0.068	−0.896 **	−0.693 **	−0.154	0.898 **	−0.443 *	0.247
	IMP	0.068	1	−0.182	−0.624 **	−0.832 **	0.312	0.657 **	0.418 *
	HxR	−0.896 **	−0.182	1	0.833 **	0.341	−0.953 **	0.211	−0.378
	Hx	−0.693 **	−0.624 **	0.833 **	1	0.787 **	−0.876 **	−0.248	−0.450 *
	XMP	−0.154	-.832 **	0.341	0.787 **	1	−0.432 *	−0.749 **	−0.347
	GMP	0.898 **	0.312	−0.953 **	−0.876 **	−0.432 *	1	−0.174	0.514 *
	UMP	−0.443 *	0.657 **	0.211	−0.248	−0.749 **	−0.174	1	0.017
	CMP	0.247	0.418 *	−0.378	−0.450 *	−0.347	0.514 *	0.017	1
−0.6	AMP	1	−0.582 **	−0.304	−0.224	0.232	0.768 **	−0.606 **	−0.322
	IMP	−0.582 **	1	0.209	−0.014	−0.172	−0.641 **	0.587 **	0.14
	HxR	−0.304	0.209	1	0.957 **	0.516 **	−0.553 **	0.552 **	−0.197
	Hx	−0.224	−0.014	0.957 **	1	0.487 *	−0.453 *	0.465 *	−0.224
	XMP	0.232	−0.172	0.516 **	0.487 *	1	−0.004	0.114	−0.453 *
	GMP	0.768 **	−0.641 **	−0.553 **	−0.453 *	−0.004	1	−0.706 **	−0.038
	UMP	−0.606 **	0.587 **	0.552 **	0.465 *	0.114	−0.706 **	1	0.023
	CMP	−0.322	0.14	−0.197	−0.224	0.453 *	−0.038	0.023	1

“*” indicating correlation at the *p* < 0.05 level and “**” indicating correlation at the *p* < 0.01 level.

**Table 3:** Effect of storage temperature on color of white mullet.

Temperature (°C)	Time (d)	L*	a*	b*
4	0	43.35 ± 0.90 ^a^	2.65 ± 0.37 ^a^	1.31 ± 0.40 ^b,c^
	1	44.19 ± 1.39 ^a^	2.32 ± 0.44 ^a,b^	2.10 ± 0.48 ^a^
	2	45.06 ± 2.78 ^a^	1.66 ± 0.85 ^a,b,c^	1.97 ± 0.30 ^a,b^
	3	44.38 ± 1.20 ^a^	1.49 ± 0.51 ^b,c^	1.54 ± 0.12 ^a,b,c^
	4	43.77 ± 4.13 ^a^	1.22 ± 0.72 ^b,c^	1.69 ± 0.06 ^a,b,c^
	5	42.07 ± 1.90 ^a^	1.14 ± 0.17 ^c^	1.40 ± 0.35 ^a,b,c^
	6	44.69 ± 0.25 ^a^	1.27 ± 0.64 ^b,c^	1.30 ± 0.51 ^b,c^
	7	46.04 ± 2.66 ^a^	1.32 ± 0.36 ^b,c^	1.21 ± 0.15 ^c^
−0.6	0	43.35 ± 0.90 ^a^	2.65 ± 0.37 ^a^	1.31 ± 0.40 ^a^
	1	44.69 ± 0.47 ^a^	2.47 ± 0.62 ^a,b^	1.95 ± 0.42 ^a^
	2	45.63 ± 2.81 ^a^	1.81 ± 0.15 ^a,b^	1.58 ± 0.33 ^a^
	3	43.28 ± 1.20 ^a^	1.51 ± 0.35 ^a,b^	1.44 ± 0.41 ^a^
	4	41.39 ± 2.55 ^a^	1.31 ± 1.16 ^b^	1.74 ± 0.17 ^a^
	5	43.82 ± 2.08 ^a^	1.96 ± 0.20 ^a,b^	1.37 ± 0.46 ^a^
	6	44.00 ± 2.91 ^a^	2.46 ± 0.31 ^a,b^	1.47 ± 1.34 ^a^
	7	45.57 ± 3.97 ^a^	2.48 ± 0.62 ^a,b^	1.39 ± 0.14 ^a^

The data are reported as the mean ± SD. The lowercase letters represent significant (*p* < 0.05) differences within the group. L*, a*, and b* represent brightness, red, and yellow, respectively.

## Data Availability

The original contributions presented in the study are included in the article, further inquiries can be directed to the corresponding author.
